# Raman flow cytometry based single‐cell species classification, viable‐cell counting and vitality test for probiotic products

**DOI:** 10.1002/imo2.70024

**Published:** 2025-05-19

**Authors:** Jia Zhang, Jianmei Wang, Pengfei Zhu, Zhidian Diao, Shuhua Tian, Ziyuan Ding, Yongming Duan, Teng Xu, Xuan Zhou, Xixian Wang, Xia Ma, Ting Sun, Xiaoyan Jing, Weilian Hung, Bo Ma, Shi Huang, Xiaowei Zheng, Jian Xu

**Affiliations:** ^1^ Single‐Cell Center, Key Laboratory of Photoelectric Conversion and Utilization of Solar Energy, Qingdao New Energy Shandong Laboratory, Qingdao Institute of Bioenergy and Bioprocess Technology Chinese Academy of Sciences Qingdao Shandong China; ^2^ Shandong Energy Institute Qingdao Shandong China; ^3^ University of Chinese Academy of Sciences Beijing China; ^4^ Qingdao Single‐Cell Biotech. Co., Ltd Qingdao Shandong China; ^5^ School of Medicine and Pharmacy Ocean University of China Qingdao China; ^6^ Nutrition & Health Research Institute COFCO Corporation Beijing China; ^7^ Inner Mongolia Dairy Technology Research Institute Co. Ltd. Hohhot China; ^8^ Yili Innovation Center, Inner Mongolia Yili Industrial Group Co. Ltd. Hohhot China; ^9^ Faculty of Dentistry The University of Hong Kong Hong Kong SAR China

**Keywords:** identification, probiotics, quality assessment, Raman flow cytometry, single‐cell Raman spectra, vitality

## Abstract

The rapidly expanding probiotic industry demands efficient methods for quality assessment of probiotic products. We previously established a microscopic imaging and Raman spectroscopy (miRS) based approach for rapid identification and tests for viability and vitality at single‐cell resolution via single‐cell Raman spectra (SCRS), yet its wider application is limited by the throughput of SCRS acquisition. Here, we employed a Raman flow cytometry (RFC) approach to accomplish this mission, which features a greatly simplified experimental workflow yet with >10‐fold higher throughput than miRS. At SCRS acquisition speed of one bacterial cell per second, RFC achieves much higher sampling depth, leading to higher accuracy of species classification than miRS in both reference ramanome database construction and quality assessment of probiotic products. Specifically, based on the fingerprint regions, the accuracy in classifying species and strains is notably improved, both for an isogenic population and for a multi‐strain probiotic product. Moreover, for probiotic products with highly biased compositions, the sensitivity of detecting and classifying low‐abundance members is order‐of‐magnitude higher. Furthermore, based on the fingerprint regions plus the C‐D band, D_2_O‐probed RFC rapidly yields viable‐cell counts and quantifies single‐cell vitality in a species/strain‐resolved fashion yet without the need for fluorescence labeling, underscoring its strength over propidium monoazide (PMA)‐based fluorescence flow cytometry. Due to its speed, accuracy, sensitivity, rich information and ease of use, RFC is a powerful platform for quality assessment for probiotics and other live‐cell products.

## Highlights

- High‐throughput and precision: Raman flow cytometry (RFC) achieves single‐cell resolution for probiotics quality‐assessment with a 10‐fold higher throughput and excellent accuracy in species classification compared to traditional methods.
- Low‐abundance detection: RFC can identify and quantify low‐abundance strains at ratios as low as 1:1000, enhancing the detection of minor components in complex probiotic products.
- Comprehensive quality control: RFC provides a label‐free, automated platform for integrated probiotics assessment, including species/strains identification, viable cell counting, and vitality measurement.

## Graphical abstract

Raman flow cytometry (RFC) enables rapid, high‐throughput quality assessment of probiotic products by classifying species/strains, counting viable cells, and quantifying vitality at single‐cell resolution. With 10‐fold higher throughput and excellent accuracy, RFC outperforms traditional methods and offers a label‐free, automated platform for probiotic quality control.

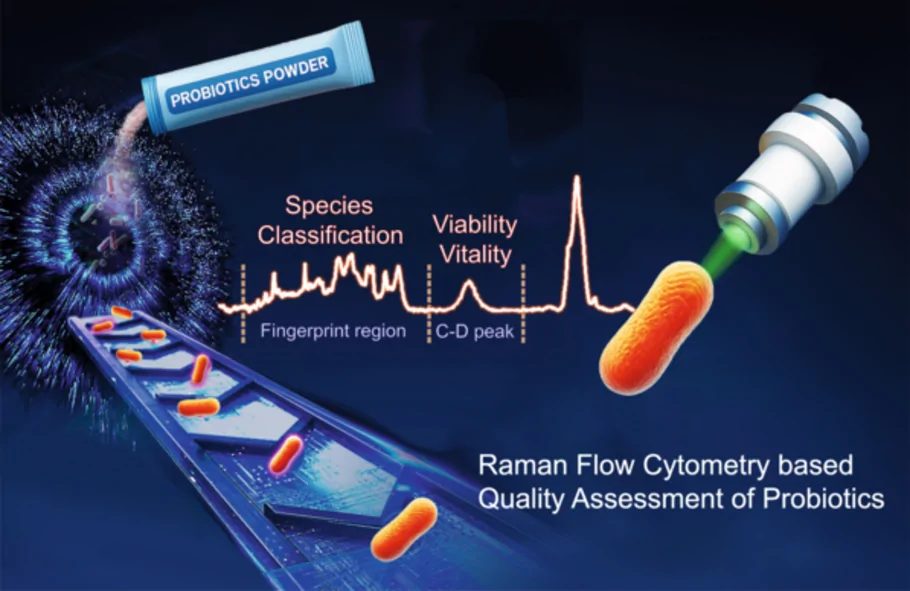

## 1 INTRODUCTION

Probiotics are defined by a consensus panel of experts in 2014 as “live microorganisms that, when administered in adequate amounts, confer a health benefit on the host” [1]. These beneficial effects have been demonstrated for treating complex symptoms and improving general health [2, 3, 4, 5]. Commercial probiotic products are available to consumers in various forms, including sachets, powders, and capsules. Regardless of the form, core quality parameters for a probiotic product, which all directly influence the product's efficacy, should encompass: (i) strain composition (i.e, the ID of each strain), (ii) total and strain‐resolved live bacterial count (viability), and (iii) total and strain‐resolved vitality. However, rapid and reliable evaluation of these core quality parameters represents grave challenges for the industry. This situation jeopardizes consumer confidence and hinders sustainable development of the field, as magnified by the revelation of product labels that carry incomplete, misleading, or even erroneous information [6, 7, 8].

First of all, for strain composition, accurate labeling of bacterial components is essential for ensuring the microbiological safety of probiotic products [9]. Given the diverse health impacts of various microorganisms, it is imperative to evaluate the “strain identification (ID)” of probiotics to ensure consistency with label claims [10]. Moreover, bacterial viability is important, as “adequate quantity” is generally one requirement for probiotics function. There is a significant correlation between health benefits and “total live‐bacteria count” consumed [11], that is, a dose–response relationship between probiotics and human health [12, 13]. For example, for managing gastrointestinal conditions including functional constipation, diarrhea, and irritable bowel syndrome, the most effective strains typically require an intake range of 10^7^ to 10^11^ CFU per day [14]. Furthermore, “vitality,” which assesses the metabolic activity and robustness of live cells in situ, is distinct from “viability,” which simply indicates whether cells are alive or dead. The vitality of probiotics is often regarded as a prerequisite for health benefits [15]. Probiotics with vitality are generally more effective than those lacking it, and the vitality and functional characteristics of microbial strains play critical roles in determining the efficacy of probiotic products [16]. Therefore, to maximize health benefits, it is essential to ensure that the formula contains sufficient quantities of specific microbial species and strains (ID), maintains adequate levels (viability), and supports metabolic activity (vitality) [17]. Together, these parameters serve as pivotal indicators for assessing the fundamental quality of probiotic products [18].

In an actual probiotic Quality Assessment (QA) process, species or strain‐resolved microbial counting, viability and vitality tests are required. However, due to the limitations of existing methods, these techniques pose significant challenges. For example, microscopic counting is unable to accurately differentiate between various species and is limited to only total bacterial counts. Plate counting requires significant manpower and resources to enumerate colonies on different selective media, and it fails to account for sub‐lethal, damaged, inhibited, dormant, or inactive cells [19]. Furthermore, the use of fluorescence in situ hybridization probes or fluorescent dyes for cell viability assessment via fluorescence microscopy or flow cytometry involves a generally slow and intricate workflow, and issues such as cell overlap may lead to the detection of only partial fluorescence signals [20, 21, 22]. Genotype‐based methods such as quantitative PCR (qPCR) are widely applied for species classification in probiotics [23]; however, they are usually unable to distinguish between live cells, dead cells and environmental DNA, and moreover, the assays can incur a substantial financial burden. Combining sequencing techniques with qPCR using ethidium monoazide bromide or propidium monoazide (PMA) enables the selective quantification of live and dead cells by inhibiting DNA amplification in nonviable cells [24, 25], however, the limitations include the inability to quantitate vitality or their intercellular heterogeneity, variability in DNA extraction efficiency (which can significantly affect the results [26, 27, 28]), high reagent costs, and operational complexity. Finally, fluorescence‐based flow cytometry usually requires suitable fluorescent dyes to label bacteria to ensure the accuracy of absolute counting, which however is influenced by factors such as choice and compatibility of dyes, bacterial size, and the settings and calibration of the flow cytometer [29]; moreover, the difficulties in designing and deploying taxonomy‐targeted or metabolism‐targeted fluorescence probes that are broadly and easily applicable have hindered the use of flow cytometry in the probiotics industry. Consequently, a methodological framework that is rapid, sensitive, accurate, comprehensive, and broadly applicable for assessing these quality parameters is a cornerstone of the probiotics industry, for both manufacturers and consumers [30].

To tackle these challenges, we have proposed a strategy called Single‐Cell Identification, Viability and Vitality Tests, and Source‐Tracking (SCIVVS) strategy for the quality inspection of probiotic products. Instead of genotype‐ or culture‐based approaches, SCIVVS employs microscopic imaging and Raman spectroscopy (miRS) to acquire single‐cell Raman Spectra (SCRS) for individual bacterial cells that are directly extracted from a probiotic product. The SCRS are analyzed for rapid classification of species (via the fingerprint regions) and for profiling the viability and vitality of the corresponding cell (via the C‐D band); when necessary, an individual cell of particular species, viability or vitality can proceed to source tracking by Raman‐activated cell‐sorting coupled to single‐cell genome sequencing [31]. However, the low‐throughput nature of miRS, which spreads cells on the surface of a dry slide, removes the medium to reduce Raman background via multiple rounds of centrifugation‐based washes, and then sequentially profiles the cells for SCRS at a speed of typically 10 s per bacterial cell, has severely limited the number of cells sampled for each probiotic population or consortium. Moreover, the shallow sampling depth, as a result of the low throughput, can directly compromise the accuracy, sensitivity and speed of probiotic QA. Current RFC technologies, such as Raman‐activated microfluidics sorting [32], Raman‐activated droplet sorting (RADS) [33] and Raman‐activated cell counting [34], are primarily limited to polydimethylsiloxane substrates. This material inherently generates strong Raman background. Moreover, Raman‐activated cell sorting (RACS) methods that adopt low‐power optical tweezers are usually unable to effectively capture fast‐moving cells, limiting their throughput to 3.3–8.3 cells per minute [13]. Furthermore, although positive dielectrophoresis (pDEP)‐based RADS (pDEP‐RADS) improves throughput, its short stable running time limits its ability to handle large cellular populations with high phenotypic heterogeneity [35]. Despite the rapid development of new image‐based, video‐based or Raman‐based flow cytometry technologies [36, 37, 38, 39], few of these platforms are capable of acquiring full‐spectrum spontaneous SCRS for bacterial cell populations while maintaining long stable running times and high throughput.

Here we employed a Raman flow cytometry (RFC) approach, based on a high‐throughput Raman flow sorter (FlowRACS) that we recently reported for label‐free, noninvasive, and high‐throughput profiling of single‐cell metabolic phenomes [40]. RFC features a greatly simplified experimental workflow yet with >10‐fold higher throughput than miRS. At SCRS acquisition speed of one bacterial cell per second, RFC achieves much higher sampling depth, leading to higher accuracy of species classification than miRS in both reference ramanome database construction and quality assessment of probiotic products. Specifically, based on the fingerprint regions, the accuracy in classification of species and strains is notably improved, both for an isogenic population and for a multi‐strain probiotic product. Moreover, for probiotic products with highly biased compositions, the sensitivity of detecting and classifying low‐abundance members is much higher. Furthermore, based on the fingerprint regions plus the C‐D band, D_2_O‐probed RFC rapidly yields viability and quantitative vitality in a fluorescence‐label‐free yet species/strain‐resolved fashion, underscoring its strength over PMA‐based Fluorescence Flow Cytometry. Due to its speed, accuracy, sensitivity, rich information and ease of use, RFC is a powerful platform for quality assessment for probiotics and other live‐cell products.

## 2 RESULTS

### Overview of the RFC strategy for quality assessment of probiotics

**Figure 1:**
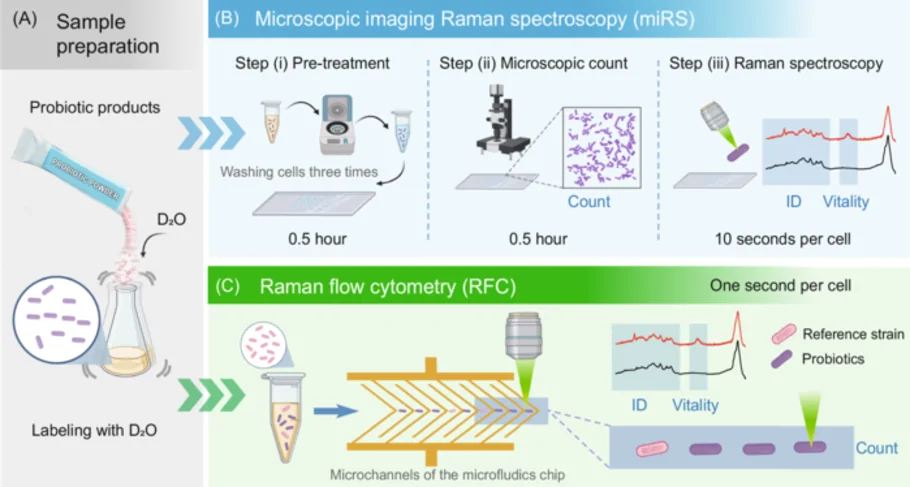
The workflow for total‐cell count, rapid identification, species‐resolved in situ viability and vitality testing of commercial probiotic products. (A) Pretreatment of probiotic powders. (B) The three‐step process for probiotic Quality Assessment (QA) based on microscopic imaging and Raman spectroscopy (miRS). (C) The one‐step process for probiotic QA using Raman flow cytometry (RFC).

The widely recognized issue with traditional probiotic quality assessment methods is their laborious and time‐consuming nature. To tackle these challenges, we have proposed a strategy called SCIVVS strategy for probiotic QA. The miRS‐based probiotic QA begins with 3 h incubation with 100% D_2_O for subsequent assessment of vitality (Figure 1A), and then unfolds in three sequential steps. (i) Pretreatment: cells are spread onto a dry slide following a series of thorough washing steps (Figure 1B). (ii) Image analysis for total‐cell count: automated image focusing, single‐cell segmentation, and localization to determine the total number of cells. (iii) Determination of ID, viability and vitality at single‐cell resolution: SCRS data is collected at a speed of about 10 s per cell, and the fingerprint regions used for identification and the C‐D band for evaluating viability and vitality [31]. Although it can complete the process in 5 h [31], this miRS approach is a slow and tedious process due to the manual nature of Step (i), the image analysis of Step (ii) and the low throughput of SCRS acquisition in Step (iii) (Figure 1B).

Another strategy to collect SCRS is via the FlowRACS system, which we have designed to concentrate and capture rapidly moving single cells within a broad channel, to facilitate efficient acquisition of SCRS and ensure prolonged operational stability [40]. Using its RFC mode, it automatically produces deeply sampled, heterogeneity‐resolved, and highly reproducible ramanome data for cellular populations or consortia of bacteria, fungus and human cells [40]. Therefore, we propose to employ RFC as a high‐throughput and automated platform for label‐free identification of probiotic species, viable cell counting, and vitality assessment at single‐cell resolution directly from a probiotic product (Figure 1C). Notably, as an internal control, a reference strain with known cell number can be incorporated into the sample for the quantification of targeted probiotics.

### Classification of species and database construction of probiotic products via RFC

The precise classification of strains is crucial in RFC. This accuracy impacts not only the identification results of probiotic products based on RFC but also the determination of total‐cell count, which is calculated by incorporating an internal reference strain with known SCRS. Consequently, the accuracy of strain classification directly influences the precision of the total‐cell count and identification in RFC.

To improve the accuracy and reliability of probiotic species identification, we should determine the best classifier, the number of spectral acquisitions, and the number of batches required for database construction. Specifically, our study focuses on the selection of the two classical species of probiotics (*Lactobacillus* species and *Bifidobacterium* species), which are widely used in industry, and collects their SCRS via RFC. Different classifiers were employed to ensure the accuracy and reliability in bacterial classification and to determine the best classifier. Moreover, we increased the number of collected SCRS to 3000 and conducted experiments in four independent batches to optimize the sampling depth and mitigate batch effects by evaluating the impact on classification accuracy.

**Figure 2:**
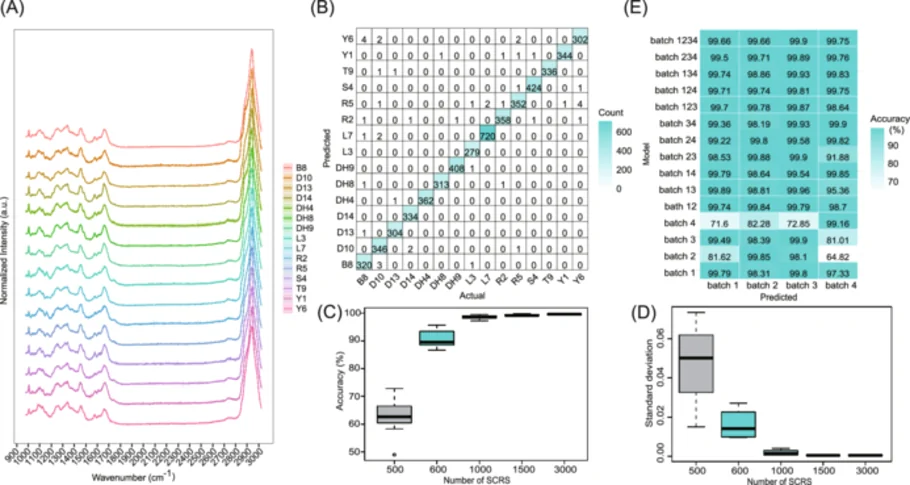
Construction of a probiotic spectral database based on RFC for species classification. (A) Average SCRS from the 15 strains of probiotics. (B) Establishment of a multi‐species Raman spectral database via LDA classifier. (C) Impact of the number of Raman spectra on classification accuracy. (D) Influence of SCRS quantity on the variability of classification accuracy. (E) Effect of the number of SCRS‐collecting batches on classification accuracy. RFC, Raman flow cytometry; SCRS, single‐cell Raman spectra.

*Step 1. Classifier determination*: Identifying the optimal classifier is crucial, as it significantly influences classification accuracy during the model and database establishment processes. Primarily, we used the RFC mode of a FlowRACS to established a Raman spectral reference database for single‐species probiotic powders, which includes 11 *Lactobacillus* species and four *Bifidobacterium* species (Table S1), providing a wealth of comparative and reference data for species classification. For each pure probiotic powder, over 1000 spectral data points were collected within 30 min (Figure 2A; Methods). After data quality control, the data set consists of 15 single‐strain probiotic powders, with a total of 18,496 valid SCRS, for constructing a multi‐species Raman spectral database (Figure 2B). To ensure accuracy and reliability in bacterial classification, six classifiers were employed. The average classification accuracy of SCRS classification using the LDA classifier reached 99.30 ± 0.05%, which is significantly higher than the performance of RF (97.31 ± 0.31%), SVM (99.17 ± 0.15%), NBC (51.10 ± 0.36%), KNN (77.57 ± 0.68%), and DT (86.00 ± 0.56%). Therefore, after three batches of repeated validation, we found the best performance in the LDA classifier (Figure 2B), which is capable of differentiating among 15 bacterial species with the highest accuracy.

*Step 2. The number of SCRS acquired*: A substantial number of SCRS enriches the information content and improves classification accuracy, but can also introduce some challenges, such as increased detection time and model redundancy. To investigate the relationship between the quantity of SCRS and classification accuracy, we selected four commonly used probiotic strains as examples: *Bifidobacterium animalis subsp.lactis* BB‐12, *Lactiplantibacillus plantarum* RH‐LP08, *Lactobacillus johnsonii* LBJ456, and *Ligilactobacillus salivarius* HH‐LS17. Briefly, we increased the number of SCRS for probiotic samples to ensure a minimum of 3000 valid spectra per sample. For independent modeling, we randomly selected 500, 600, 1000, 1500, and 3000 spectra, conducting five repetitions for each selection. The data were split into 70% for model training and 30% for predictive analysis. The predictive classification accuracy for randomly selecting 500 spectra (63.39 ± 4.72%), 600 spectra (90.59 ± 2.70%) or 1000 spectra (98.53 ± 0.18%) was significantly lower than that for 1500 spectra (99.26 ± 0.04%) and 3000 spectra (99.59 ± 0.05%) (Figure 2C). As the number of spectra approached a threshold, the growth rate of classification accuracy began to stabilize (Figure 2C). As the sample size increases, the standard deviation (SD) of predictive classification accuracy shows a declining trend. Specifically, the SD values for random selections of 500, 600, and 1000 spectra were 4.72%, 2.70%, and 0.18%, respectively. In marked contrast, the SD values for 1500 and 3000 spectra were significantly lower, at 0.04% and 0.05% (Figure 2D). Previous studies have suggested a minimum sampling depth of approximately 50–200 SCRS for adequate representation [35, 39, 40], which is evidently insufficient for analyzing complex microbial samples. As the sampling depth increases, both classification accuracy and volatility tend to stabilize around 1500 spectra. This nonlinear relationship underscores the impact of data quantity on model performance, indicating a threshold effect of spectral quantity on classification accuracy. Therefore, a data set comprising at least 1500 spectra is essential for constructing a robust database that optimizes classification accuracy and maintains model robustness. RFC has the capability to measure 1500 spectra in approximately 30 min, compared to about 4 h required by miRS. This illustrates a substantial time advantage, making RFC particularly beneficial for high‐throughput analysis and rapid detection of probiotic quality evaluation.

*Step 3. The number of batches*: To ensure the reliability and reproducibility of species classification results, we collected SCRS of the aforementioned four commonly used probiotic strains across four independent batches to investigate the impact of batch variations on species classification accuracy. Randomly selection of over 1500 spectral data points from a single batch for database construction yielded a species classification accuracy of 99.68 ± 0.35% (with the accuracy of miRS accuracy being 93.02%; [31]). This accuracy was significantly higher than that of species classification predictions based on data from other batches, which averaged 87.13 ± 12.89% (Figure 2E). Thus, the classification model built using a single batch of data would be limited in its general applicability, and batch effects can significantly impact the accuracy of species classification results. To address this issue, we subsequently selected over 1500 spectral data points from two, three, or four batches, respectively, for database construction to predict species classification results from all batches. To ensure the robustness and general applicability of our model, we conducted cross‐validation across four batches that were independently prepared and measured on different days with varying experimental conditions such as temperature, humidity, and instrument performance, respectively (Methods). Specifically, by training on two batches and testing on another independent batch, we confirmed that our classifiers can maintain a high accuracy of 98.83 ± 2.26% (Figure 2E), which supports their ability to generalize across different experimental sessions. Furthermore, the average accuracy of species classification for other batches using databases constructed from three or four batches was 99.64 ± 0.36% and 99.74 ± 0.11%, respectively, showing no statistically significant difference between the results obtained from the two‐batch and multi‐batch constructions (Figure 2E). These findings indicate that combining data from two or more batches for database construction effectively mitigates the impact of batch effects on classification accuracy, enhancing the generalization capability of the model and improving the reliability and reproducibility of species classification. To summarize, for improved accuracy and reliability in identifying probiotic species, the LDA classifier is optimal, necessitating at least 1500 spectra for robust accuracy, while multi‐batch data further enhances reliability.

Moreover, to verify the ability of our analysis method to classify 15 strains and estimate the concentration of each, we extracted 60 Raman spectra per strain from the test set, yielding a total of 900 Raman spectra consisting of 15 strains mixed in equal proportions. This process was repeated three times, and the same model was applied to predict the mixed data. The prediction results showed no significant difference between the true proportion of the 15 strains and the results obtained from our prediction (Figure S1). This demonstrated the potential of our method in accurately classifying and quantifying the 15 bacterial strains from a mixture.

### Classification of strains in mock probiotic products via RFC

**Figure 3:**
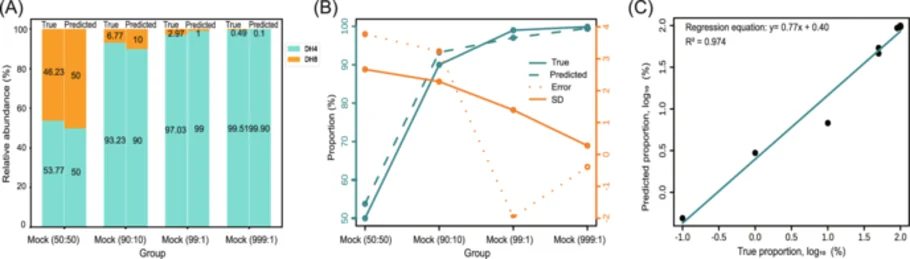
Species identification of mock probiotic samples. (A) Performance comparison of predicted versus true relative abundance of mock samples with varying ratios. (B) Proportion and error analysis of predicted versus true relative abundances of mock samples. (C) Correlation analysis between predicted and true relative abundances of mock samples.

To test the accuracy of LDA model on species ID, we started with a mock microbiota by mixing *B. lactis* BB‐12 and *L. plantarum* RH‐LP08, which are widely used and commercially available probiotic strains [41, 42], in ratios of 1:1, 9:1, 99:1, and 999:1 (Figure 3A). For each mock probiotic sample, over 1500 randomized spectral data‐based identification experiments were conducted using the LDA classifier. In each experiment, the proportions of *B. lactis* BB‐12 and *L. plantarum* RH‐LP08 in the mock communities were accurately reconstructed, with discrepancies between predicted and actual results being <3.77% (Figure 3B). Thus, the model reliably distinguishes single cells of *B. lactis* BB‐12 from those of *L. plantarum* RH‐LP08 in the mock probiotic products.

Notably, even when the relative abundance of *B. lactis* BB‐12 is as low as 1:1000, RFC is capable of achieving accurate detection of such low relative abundance species, when increasing the depth of SCRS sampling to 5000. RFC takes approximately 1.5 h to obtain 5000 SCRS, while miRS requires over 10 h due to its detection speed of 10 seconds per cell. A significant positive correlation was observed between the actual and predicted proportions of *B. lactis* BB‐12 and *L. plantarum* RH‐LP08 (*R* ^2^ = 0.974; Figure 3C). In summary, RFC has demonstrated its utility as a technique for species identification and evaluation of complex probiotic products. Its analytical depth is 10 times greater than that of miRS, enhancing its applicability in probiotic quality control processes. For instance, RFC can accurately identify and quantify probiotic strains present at ratios as low as 1:1000, while miRS is unable to reliably detect such minor components.

Furthermore, RFC demonstrated remarkable efficacy in accurately distinguishing between different strains of the same species, an endeavor that presents significant challenges for traditional methods. Specifically, *Lactobacillus* species have high commercial value, serving as the most widely used species in the probiotic industry [43]. In addition, the genotypes of *L. paracasei* YL16 and *L. paracasei* YL17 are so similar that even costly and time‐consuming sequencing cannot distinguish between them. In this study, *L. paracasei* YL16 and *L. paracasei* YL17 were used to establish a reference SCRS database. To test identification effectiveness, we mixed *L. paracasei* YL16 and *L. paracasei* YL17 in ratios of 1:1 and 10:1, followed by RFC detection of the mock probiotic samples. For each mock sample, over 1500 randomized SCRS‐based classification experiments were conducted using the LDA classifier. In each experiment, the proportions of *L. paracasei* YL16 and *L. paracasei* YL17 in the mock community were accurately reconstructed, with discrepancies between predicted and actual results remaining below 3.32% (Figure S2). Thus, the model reliably distinguished between *L. paracasei* YL16 and *L. paracasei* YL17 cells, demonstrating that RFC can effectively distinguish between different strains of the same species. Raman spectroscopy detects laser‐excited Raman scattered light, revealing molecular vibrational modes through wavenumber shifts. This non‐destructive technique provides information‐rich, fingerprint‐like spectra of intracellular biomolecules (e.g., proteins, nucleic acids, lipids, polysaccharides), enabling label‐free, simultaneous profiling of viability, vitality, and a variety of metabolic phenotypes [44, 45, 46]. Moreover, differences in macromolecule content and chemical band vibrations among species can manifest as distinct Raman peak shifts and intensity variations, potentially serving as molecular identifiers for species classification [47, 48]. In contrast, fluorescence‐based flow cytometry, which relies on fluorescent dyes or probes that label DNA, proteins or other types of metabolites for its target specificity, is limited by information content, and usually unable to provide species, viability and vitality all together [49, 50].

### Total‐cell count in probiotic products based on species classification via RFC

**Figure 4:**
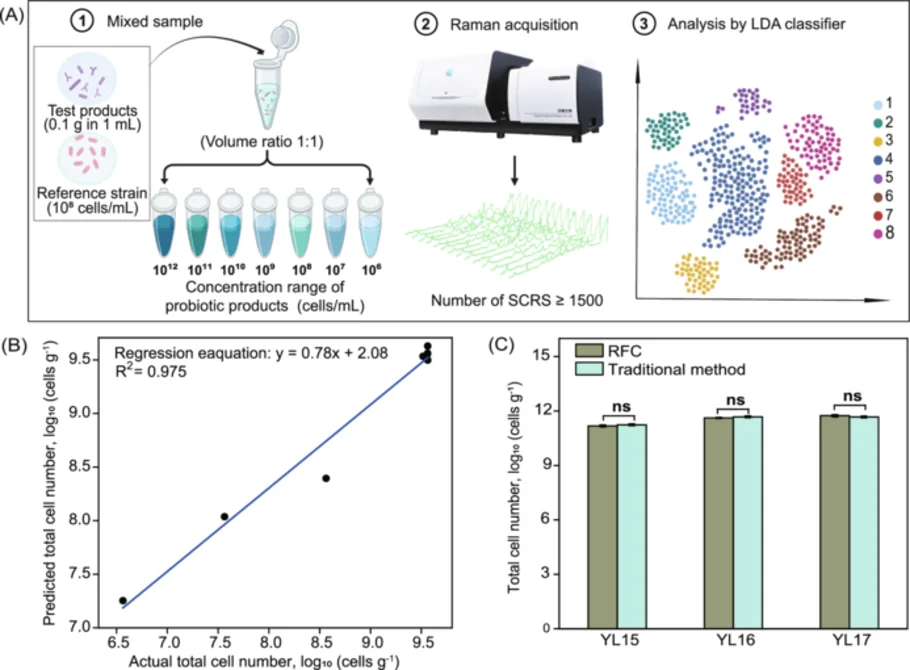
Raman flow cytometry (RFC)‐based total cell counting of probiotics at single‐cell resolution. (A) Methodological workflow for RFC counting and applicable product cell content range. ① Mixing test products and reference strains according to the ratio indicated in the figure. The concentrations of test samples range from 10^6^ to 10^12^ cells/g. ② Collecting over 1500 SCRS from the mixed samples. ③ Determining the ratio of mixed strains using the LDA classifier and calculate the total‐cell count of the products based on reference strains of the predicted ratio. (B) Correlation of RFC‐based and true cell numbers of mock samples. (C) Total‐cell count of probiotic products using RFC versus the traditional counting method. SCRS, single‐cell Raman spectra.

Profiling probiotic products using RFC in a high‐speed flowing liquid phase poses challenges for directly counting the total number of cells based on SCRS. To mitigate this challenge, we introduced a known quantity of a reference strain and mixed it with the test strains at a specific ratio. By applying the species identification method illustrated in this study, we were able to determine the relative ratios of the two strains. This, in turn, enabled us to calculate the absolute quantity of the test strains based on the known quantity of the reference strains (Figure 4A).

To evaluate the detection limits of our approach for directly counting the total number of cells, we designated one strain as the reference strain and the other as the test strain, mixing them in a specific ratio (Figure 4A) for absolute cell count. Before mixing, we determined the total cell counts of both strains using the hemocytometer counting method. Utilizing the classification results (Figure 3), we accurately predicted the mixing ratios. Thus, our method can accurately measure total‐cell count, even the concentration ratio of the reference strain to the test strain is as high as 1000‐fold. Moreover, the actual and predicted cell numbers are highly correlated (*R* ^2^ = 0.975; Figure 4B). Therefore, when enumerating cells in probiotic products, we recommend mixing in a reference strain at an appropriate order of magnitude based on the stated strain counts in the product specifications (Figure 4A). Thus, the calibration method ensures reliable and accurate quantification by changing the cells number of reference strains.

To assess the accuracy and applicability of the aforementioned methods, we selected three single‐strain probiotic powder products for testing, by employing traditional methods for a parallel comparison. A known quantity of *Escherichia coli* was utilized as a reference strain and was mixed separately with the single‐strain probiotic powder products for RFC detection. The relative abundances of *B. animalis* YL15, *L. paracasei* YL16, and *L. paracasei* YL17 in these single‐strain probiotic products were predicted by RFC. As a result, the total bacterial counts in the different products were as follows: *B. animalis* YL15: 1.74 ± 0.20 × 10^11^ CFU/g, *L. paracasei* YL16: 4.92 ± 0.56 × 10^11^ CFU/g; *L. paracasei* YL17: 4.73 ± 0.54 × 10^11^ CFU/g. Importantly, these counts showed no significant difference compared to traditional counting methods (*p* > 0.05, Figure 4C). Therefore, RFC can perform accurate total‐cell counting directly from probiotic products in a culture‐free, fully automated and single‐cell‐resolved fashion.

### Measurement of probiotic viability, vitality, and cellular heterogeneity via RFC

Ramanome, as a kind of single‐cell‐resolution metabolic phenome, has demonstrated the ability of SCRS to rapidly classify microbial species by fingerprint regions and quantitatively measure metabolic viability and vitality by C‐D peaks (via tracking D_2_O intake) [51, 52]. Therefore, RFC is capable of performing high‐throughput analysis of species/strain‐specific single‐cell viability and vitality profiles in probiotic products. When cells are incubated with isotope‐labeled substrates such as D_2_O, the metabolism of such substrates would induce characteristic Raman peak shifts, which can serve as a simple and quantitative biomarker for cellular viability and vitality. Specifically, in the presence of D_2_O, the exchange of hydrogen ions (H^+^) from intracellular electron carriers such as NADPH+H^+^ and NADH+H^+^ with deuterium ions (D^+^) from D_2_O would ensue in viable cells, eventually leading to the progressive replacement of C‐H bands by C‐D bands in intracellular macromolecules like lipids and carbohydrates [53, 54, 55]. Moreover, the rate of deuterium incorporation into individual living cells via NADPH/NADH, which is proportional to the metabolic vitality of the cell, can be quantified by monitoring the shift from the C‐H band to the C‐D band in SCRS [56]. Given that the uptake of H_2_O or D_2_O is a universal feature of living cells, D_2_O‐based single‐cell Raman microspectroscopy can serve as a noninvasive, quantitative, and universal method for detecting and quantifying the viability as well as metabolic activity of a cell [56, 57]. Incubation with 100% D_2_O for 3 h has a significant inhibitory effect on bacterial growth, as bacterial cell division is inhibited in the 100% D_2_O environment, and this is important to ensure the accuracy of live‐bacteria counts after the incubation [31]. Notably, although growth is fully inhibited by 100% D_2_O, a short‐term incubation time of 3 h would not inhibit the uptake of D_2_O and would still result in rapid uptake of D_2_O (Figure 4C; [53, 58]). Therefore, we selected the metabolic activity level (MAL) after 3 h of incubation with 100% D_2_O as the default criterion for viability rate counting based on SCRS [31], with MAL ≤ 0 indicating dead cells and MAL > 0 indicating live cells. Beyond the viability‐based counting, the “in situ vitality” of individual cells can also be directly derived from the SCRS sampled from probiotic products. The “in situ vitality” serves as a valuable indicator for assessing the metabolic activity of microorganisms. Furthermore, the degree of intercellular heterogeneity in vitality can be quantified using MAL‐HI.

**Figure 5:**
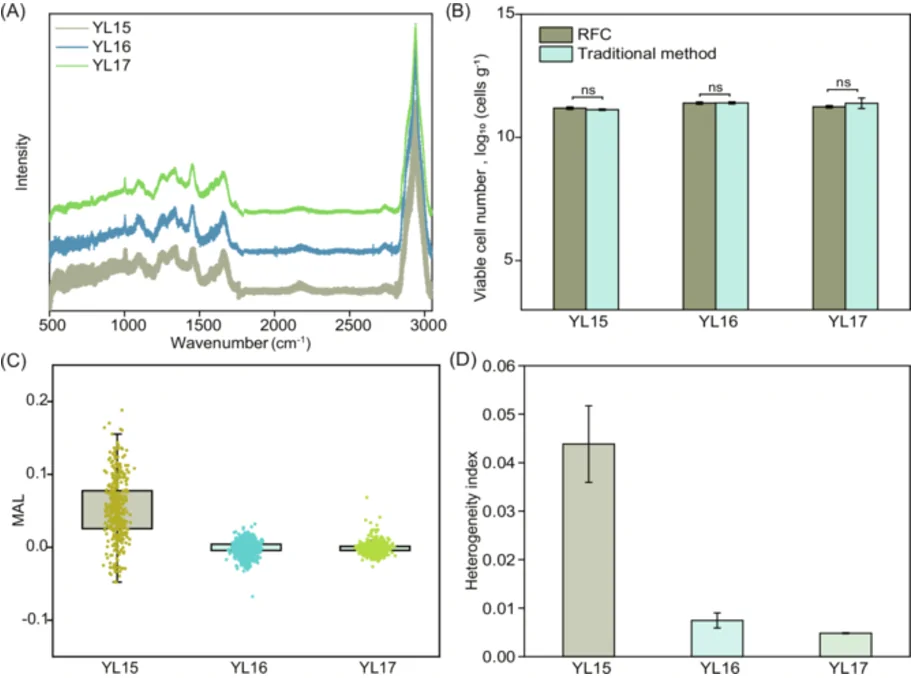
Raman flow cytometry (RFC)‐based in situ vitality, viability and cellular heterogeneity tests of probiotics at single‐cell resolution. (A) Average single‐cell Raman spectra (SCRS) from three probiotic powder products, displayed in bold and overlaid on representative examples of SCRS for each strain. (B) In situ viability assessment based on the RFC method compared to traditional counting method. (C) SCRS‐based in situ metabolic activity level (MAL) tests of probiotics at single‐cell resolution. (D) In situ MAL‐HI from the three probiotic powder products.

Specifically, we selected three single‐strain probiotic powder products (*B. animalis* YL15, *L. paracasei* YL16, and *L. paracasei* YL17) for the testing, while employing traditional methods for parallel comparison. For each strain studied, over 1500 SCRS were obtained (Figure 5A). The viability rates calculated for *B. animalis* YL15 (89.95 ± 4.02%), *L. paracasei* YL16 (50.43 ± 5.82%), and *L. paracasei* YL17 (36.98 ± 4.73%) via RFC were not significantly different from the viability rates determined by traditional counting methods (Student's *t*‐test: *p* > 0.05; Table S2), confirming the utility of RFC for assessing the viability rates of probiotic products.

Based on the total‐cell count results presented in Figure 4C, the predicted viable‐cell counts for different species using the RFC method were comparable to those from traditional methods. For *B. animalis* YL15, the RFC method yielded 1.54 ± 0.18 × 10^11^ CFU/g, versus 1.34 ± 0.11 × 10^11^ CFU/g with the traditional method, showing no significant difference (*p* = 0.24 via Student's *t*‐test). For *L. paracasei* YL16, the RFC method gave 2.48 ± 0.28 × 10^11^ CFU/g, and the traditional method gave 2.70 ± 0.31 × 10^11^ CFU/g, again with no significant difference (*p* = 0.86 via Student's *t*‐test). For *L. paracasei* YL17, the RFC method had a median of 1.75 × 10^11^ CFU/g, and the traditional method had a median of 3.10 × 10^11^ CFU/g, with no significant difference (*p* = 0.66 via Mann–Whitney *U* test). Overall, statistical analyses using Student's *t*‐test and Mann–Whitney *U* test confirmed no significant differences between the two methods (*p* > 0.05; Figure 5B). Thus, RFC demonstrates the capability to perform accurate live‐cell count directly from probiotic products in a culture‐free manner.

Notably, the average MAL, representing “in situ vitality,” for the *B. animalis* YL15 product was 0.0528 ± 0.0113, which was significantly higher than those of the *L. paracasei* YL16 (Student's *t*‐test; *p* = 0.02) and *L. paracasei* YL17 (Student's *t*‐test; *p* = 0.02) products (Figure 5C). Furthermore, the MAL‐HI of the *B. animalis* YL15 product was greater than that of the *L. paracasei* YL16 and *L. paracasei* YL17 products, indicating that the metabolic vitality of individual *B. animalis* YL15 cells was not only higher on average but also more uniform (Figure 5D). To validate the measurement precision of our method under specific equipment and collection conditions, we performed experiments using *Escherichia coli* samples with C‐D ratio (CDR) values of 0 and 0.06, respectively. Each sample was measured three times under varying energy and time conditions. Our results suggest that when measurement conditions exceed 300 mW for 1 s, the coefficient of variation (CV) for both CDR values reaches its minimum, at 0.2279 ± 0.0147 and 0.0476 ± 0.0031, respectively (Figure S3). These findings suggest that the error introduced by our equipment and collection conditions is consistently maintained at a very low level, which ensures reliable and reproducible results.

Consequently, for probiotic products with similar live‐cell count, RFC can rapidly distinguish not only the levels of metabolic vitality but also their heterogeneity, reflecting the degree of synchronization among cells.

### An automated, sensitive and integrated RFC workflow for multi‐strain probiotic products quality control

To comprehensively verify the robust application of RFC technology in probiotic QA, we validated the integrated RFC workflow on commercial multi‐strain probiotic products, utilizing the BKE3 product (Table S1) from Yili Group as a case study. The probiotic product BKE3 is a multi‐strain formulation that includes three specific strains: *L. paracasei* K56, *L. paracasei* ET‐22, and *B. animalis* BL‐99. Most commercially available probiotic solid beverages predominantly feature strains from *Bifidobacterium spp*. and *Lactobacillus spp*. Remarkably, BKE3 integrates representative strains from both of these genera. Additionally, the presence of multiple strains from the same species further complicates its analysis, making BKE3 a challenging candidate for demonstration. Notably, traditional methods for quality inspection of multi‐strain probiotic products, particularly those consisting of different strains from the same species, often fall short in delivering comprehensive quality evaluations. In contrast, the RFC technology employed in this study integrates multiple indicators, enabling a thorough quality inspection of such multi‐strain probiotic products.

**Figure 6:**
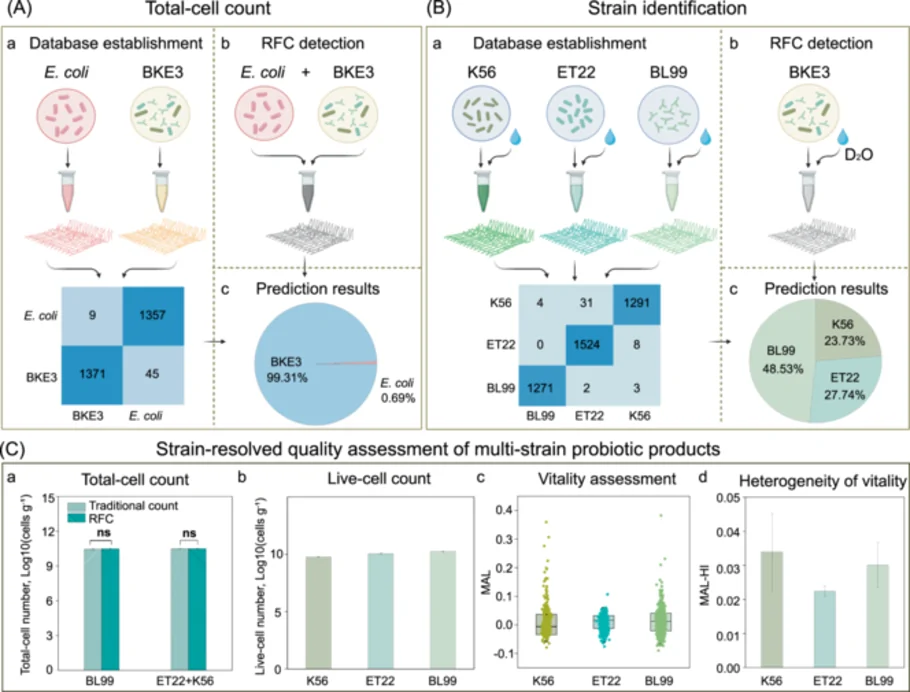
An integrated single‐cell Raman flow cytometry (RFC) workflow for multi‐strain probiotic Quality Assessment (QA). (A) Total‐cell count of a multi‐strain probiotic product (BKE3) by RFC. (a) Collecting the single‐cell Raman spectra (SCRS) of the BKE3 and the reference strain (*E. coli*) separately for database establishment. (b) Collecting the SCRS of BKE3, which was co‐tested with *E. coli* by RFC. (c) The predicted ratio of BKE3 and *E. coli* through the LDA classifier via SCRS. (B) Strain identification of BKE3 by RFC. (a) Incubating the cells from three pure probiotic powders (*L. paracasei* K56, *L. paracasei* ET‐22, and *B. animalis* BL‐99) with 100% D_2_O for 3 h and collecting SCRS separately for database establishment. (b) Incubating BKE3 with 100% D_2_O for 3 h and collecting the SCRS by RFC. (c) Identifying the predicted proportions of three strains from BKE3. (C) Strain‐resolved quality assessment of multi‐strain probiotic products. (a) Total‐cell counts of species in BKE3 as assessed by the traditional methods and by RFC. (b) Live‐cell counts of each strain in BKE3 as assessed by RFC. (c) The metabolic activity level (MAL) of each strain in BKE3 as measured by RFC. (d) The MAL‐HI of each strain in BKE3 as measured by RFC.

*Step 1: Total‐cell count:* We employed a method that involves introducing a known quantity of reference strain for quantifying probiotic products (Figure 6A). Initially, we collected spectra of the multi‐strain probiotic product BKE3 and the reference strain *E. coli* separately, ensuring at least two batches for each sample and obtaining 1500 valid spectra per batch for database classification (Figure 6A‐a). Subsequently, *E. coli* was co‐analyzed with BKE3 using RFC to determine the relative proportions by LDA classifier. The known quantity of the reference strain *E. coli* was 4.87 ± 0.13 × 10^8^ CFU/mL. The test product and reference strain were mixed in equal volumes, yielding an actual cell number ratio of *E. coli* to BKE3 of 1:137.71 (Figure 6A,B). Based on calculations, the predicted ratio of *E. coli* to product BKE3 obtained through the LDA classifier was 1:143.48 (Figure 6A–C). Therefore, the total‐cell count of product BKE3 was predicted as 6.15 ± 0.70 × 10^10^ CFU/g, which showed no statistically significant difference from the actual total‐cell count of the product (5.73 ± 1.12 × 10^10^ CFU/g; *p* > 0.05) as determined by the *t*‐test. Consequently, RFC can accurately predict the total‐cell count of the multi‐strain probiotic product BKE3.

*Step 2: Strain identification:* We constructed a reference SCRS database from the three ingredient strains in BKE3: *L. paracasei* K56, *L. paracasei* ET‐22, and *B. animalis* BL‐99. Cells from each pure probiotic powder were incubated with 100% D_2_O for 3 h, and the SCRS were acquired using a 532 nm laser (Figure 6B‐a). Performance analysis showed that the average classification accuracy of the SCRS‐based method for distinguishing among the three classes was 98.74% (Figure 6B‐a). This high level of accuracy indicates the model's reliable capacity to differentiate among the strains present in BKE3.

To further evaluate the feasibility of direct strain identification from the products, BKE3 was incubated with 100% D_2_O for 3 h, and approximately 1500 SCRS were acquired directly from the mixture of cells derived from BKE3 (Figure 6B‐b). Using the previously established LDA classifier for the SCRS‐based identification of each probiotic cell sample, we categorized the three strains from BKE3 into separate files. Then, the predicted proportions of total cells within BKE3 were identified as follows: *B. animalis* BL‐99 at 48.53 ± 0.61%, *L. paracasei* ET‐22 at 27.74 ± 0.90%, and *L. paracase*i K56 at 23.73 ± 0.52% (Figure 6B,C). However, using traditional 16S rDNA amplicon sequencing, only species‐level total‐cell proportions can be determined. *B. animalis* BL‐99 constituted 47.95 ± 3.56%, while *L. paracasei*, encompassing both *L. paracasei* K56 and *L. paracasei* ET‐22, accounted for 51.19 ± 4.10%. Thus, according to RFC analysis, the total‐cell counts for the two species in BKE3 were *L. paracasei* at 3.17 ± 0.37 × 10^10^ CFU/g and *B. animalis* at 2.99 ± 0.34 × 10^10^ CFU/g. On the other hand, 16S rDNA amplicon sequencing revealed the total‐cell counts in BKE3 as *L. paracasei* at 3.12 ± 0.17 × 10^10^ CFU/g and *B. animalis* at 2.97 ± 0.53 × 10^10^ CFU/g. The results did not differ significantly (*t*‐test; *p* > 0.05; Figure 6C‐a). Thus, the SCRS‐based strain‐level classification in BKE3 was accurate. Notably, as traditional methods often struggle to achieve precise results for different strains of the same species in multi‐strain probiotic products [59], this SCRS‐based method via RFC provides a viable solution to this challenge.

*Step 3: Strain‐resolved live‐cell count, vitality assessment, and heterogeneity of vitality:* Within each strain‐specific file, we determined the SCRS‐based viability rates (MAL ≤ 0 for dead cells and MAL > 0 for live cells) for each strain: *L. paracasei* K56 at 40.20 ± 7.71%, *L. paracasei* ET‐22 at 68.37 ± 7.85%, and *B. animalis* BL‐99 at 58.24 ± 13.08%. Using these viability rates and the total‐cell counts, we calculated the live‐cell count for each strain as follows: *L. paracasei* K56 at 5.86 ± 0.55 × 10^9^ CFU/g, *L. paracasei* ET‐22 at 1.17 ± 0.16 × 10^10^ CFU/g, and *B. animalis* BL‐99 at 1.74 ± 0.20 × 10^10^ CFU/g (Figure 6C‐b).

Moreover, the average MAL values for *B. animalis* BL‐99, *L. paracasei* ET‐22, and *L. paracasei* K56 were found to be 0.0099 ± 0.0015, 0.0098 ± 0.0011, and 0.0014 ± 0.0007, respectively (Figure 6C‐c). The MAL‐HI for these strains were 0.0301 ± 0.0066, 0.0224 ± 0.0015, and 0.0339 ± 0.0113 (Figure 6C,D), highlighting the variations in metabolic vitality among individual cells across different species. Therefore, RFC enables the integrated detection of total‐cell count, strain identification, viability assessment, metabolic activity quantification, and evaluation of vitality heterogeneity in multi‐strain probiotic products.

Considering the estimated duration of experiments and the cost of consumables, the RFC process takes approximately 3.5 h and costs $4.39 for comprehensive probiotic QA, which includes live‐cell counting, strain identification, and species resolved in situ viability and vitality analysis. In comparison, the miRS process takes about 5 h and incurs a similar cost to RFC [31]. Traditional methods, however, require 9–11 days and incur costs ranging from $33.31 to $58.61 for live‐cell counts, strain identification, and vitality testing (via cytometry) [31]. As a result, the RFC method is over 20 times faster than traditional approaches and provides a detection limit that is over 10 times lower than miRS (Table 1).

**Table 1:** Comparison of quality assessment methods for probiotic products: Microscopic imaging and Raman spectroscopy (miRS) versus Raman flow cytometry (RFC).

	miRS	RFC
Instrument	RACS‐Seq	FlowRACS
Throughput of SCRS acquisition	~10 seconds per cell	~One second per cell
Operation	Three steps (cell washing, microscopic imaging, SCRS acquisition)	One step (SCRS acquisition)
Accuracy of strain classification (for the example cited in results)	93.02 ± 1.39%	99.68 ± 0.35%
Detection limit (for the example cited in results)	1/100	1/1000

Abbreviations: miRS, microscopic imaging and Raman spectroscopy; RFC, Raman flow cytometry; SCRS, single‐cell Raman spectra.

## 3 DISCUSSION

As a cell is the basic unit of function and the atomic step of evolution for a probiotic product, a platform that can rapidly and label‐freely perform total‐cell count, classification, viability test and vitality measurement at single‐cell resolution represents the ultimate capability for probiotic QA. The SCIVVS strategy can fulfill this promise, yet its present implementation of miRS, which is based on microscopic bright‐field imaging for single‐cell counting and profiling of SCRS for cells spread out on the surface of a dry slide, is limited by the throughput of SCRS acquisition. The RFC implementation of SCIVVS can improve the throughput by over 10‐fold, by saving the multiple rounds of centrifugation‐based washing and via the FlowRACS system that employs the Positive Dielectrophoresis‐Induced Deterministic Lateral Displacement technology for SCRS‐based flow cytometry [40]. As a result, RFC, based on FlowRACS, offers multiple advantages in probiotic QA (Table 1). (i) Higher accuracy in species classification: the strain classification accuracy via RFC can reach as high as 99.68 ± 0.35%, compared to those below 95% in miRS‐based studies [51, 52]. Additionally, when batch efforts were taken into consideration, RFC can reach an average classification accuracy of 98.83%. (ii) Higher sensitivity in detecting low‐abundance members of consortium: due to its much higher sampling depth within a given timeframe, RFC can reach a detection limit at least 10 times lower than miRS [31, 60] or the traditional plate methods [61]. (iii) Proneness to automation: in RFC, probiotic cells were counted for both absolute and relative abundance while simultaneously profiled for SCRS in a fully aqueous phase. This automated process avoids the tedious and manual operations in miRS that include sampling washing via centrifugation, spreading cells on a slide, etc. This feature also suggests RFC can be extended to real‐time, continuous monitoring of a mixed‐culture based fermentation process for probiotic QA. In particular, as compared to current methods such as qPCR or fluorescence‐based flow cytometry, RFC offers the advantages of directly and simultaneously providing species‐resolved viability, vitality and intercellular metabolic heterogeneity yet without the need for expensive reagents or complex experimental procedures.

Notably, the higher sampling depth enabled by the elevated throughput has important implications for species/strain ID and classification. Our results suggest that the higher sampling depth and incorporation of data from multiple batches would lead to the construction of reference ramanome databases that achieved higher accuracy in classification, higher robustness in models, and improved ability to distinguish among multiple strains from a single species. Therefore, for the construction of such reference ramanome databases for SCRS‐based ID or classification directly from a clinical or environmental microbiota, RFC would be the preferred approach over miRS. As for the throughput of handling samples, a single instrument can process up to 48 samples in tandem, with continuous 24‐h by one technician within a day (or 16 samples in eight working hours for one technician), assuming a turnaround time of 0.5 h per sample. When testing demand increases, (i) for instrument parameters, the turn‐around time for each sample can be reduced by half by elevating the flow rate in RFC to 2–3 cells/second through enhanced hydrodynamic focusing and laser triggering synchronization, while multi‐lane microfluidic chips for RFC can enable parallel processing of four to eight samples simultaneously; (ii) for personnel allocation, development of automated sample loading and AI‐assisted data analyses would reduce manual intervention, allowing one technician to oversee 3 to 5 instruments concurrently; (iii) for consumable expense, the costs can be controlled, since the RFC chips are reusable after washing, while the amount of D_2_O consumed for each sample can be as low as <100 microliter. Therefore, with such adjustments, the RFC approach can be scaled up to meet the demands of high sample throughput like those in the probiotics industry.

Despite its strengths, further developments are necessary to fully realize the potential of RFC for probiotics QA. (i) Tackling cellular adhesion due to exopolysaccharides: certain probiotic strains produce exopolysaccharides [62], which can cause the adherence of bacterial cells to the flow channel of the RFC chip during the process of SCRS acquisition. Additional efforts, such as altering buffer formulations or adjusting the design of current RFC chips, may be necessary to tackle probiotic products that contain such strains [63]. (ii) Expanding the scope of applicable commercial products: commercial probiotic products can exhibit a higher degree of complexity than what was demonstrated in this study, due to the mixture of more strains or the use of additional matrix components. Although they can potentially affect the accuracy of testing results, these confounding factors can be tackled by testing our approach across a broader range of products, optimizing the sample pretreatment procedure, expanding the reference databases of probiotics strains, or refining the classification algorithms. (iii) Recognition of unknown species: our approach requires prior training on reference spectral data for species identification. Although this requirement can be accommodated in a typical probiotics QA setting where members of the product are generally known and well‐characterized, classification of unknown species can pose a challenge. To tackle this limitation, algorithms for anomaly detection or transfer learning that fine‐tune model parameters with limited spectral data from new strains should be developed, so as to enhance accuracy in classifying or identifying new species while reducing the costs of expanding the reference database of Raman spectra [64, 65]. (iv) Consideration of a broader range of metabolic phenotypes: each SCRS collected by RFC contains rich spectral information that can reveal a broader range of metabolic phenotypes, such as substrate intake [44], product profile [66], drug resistance [67], intracellular conversion among metabolites [68], and cross‐feeding among cells [51, 54]. Incorporating these additional single‐cell‐resolved metabolic features into probiotic QA would be a fruitful direction for development. (v) Expansion from Raman profiling to Raman‐activated cell sorting: in many circumstance, validation and thorough analyses of cells of interest would require sorting followed by genome sequencing or cultivation, therefore developing a workflow that allows both profiling and sorting of cells based on species, viability, or vitality would further widen the application of this approach in the probiotic industry [69, 70, 71]. (vi) Cautious interpretation of low‐vitality strains: for such strains, future experiments should focus on optimizing the D_2_O incubation conditions, including concentration and time, to ensure that the CDR signal generated by the strain is sufficient to distinguish between live and dead cells and quantify vitality, while also confirming that the strain exhibits no significant changes in abundance during D_2_O incubation. This optimization will help validate the reliability of the method for assessing the viability and vitality of low‐vitality strains.

## 4 CONCLUSION

To develop a rapid and efficient method for comprehensive and in‐depth probiotic QA, we introduced an RFC approach that features a greatly simplified experimental workflow yet with >10‐fold higher throughput in acquisition of SCRS than miRS. The advantage in throughput allows higher accuracy of species classification both in reference ramanome database construction and in probiotic QA, due to the much higher sampling depth. Moreover, the sensitivity of detecting and classifying low‐abundance members is greatly elevated. Due to its speed, accuracy, sensitivity, rich information, and ease of use, RFC is a powerful platform for quality assessment for probiotics and other live‐cell products.

## 5 METHODS

### Probiotic strains and products

In this study, 19 probiotic products in total were tested (Table S1). All strains were from Mengniu Dairy Co. Ltd, Renhe Pharmacy Co. Ltd, and Zhengzhou H&H Bioengineering Co. Ltd.

### Operation of RFC using a FlowRACS instrument

FlowRACS, designed for high‐throughput single‐cell metabolic phenome profiling, is comprised of an automated flow‐based Raman platform and a microfluidic chip fabricated from quartz [36, 37]; (Qingdao Single‐cell Biotech, CN). The chip is bonded with a bottom Indium tin oxide (ITO)‐electrode array layer and a top thin cover slice using double‐sided tape. The system functions by utilizing a positive dielectrophoresis‐induced deterministic lateral displacement (pDEP‐DLD) force to focus and trap individual cells, facilitating the efficient acquisition of full‐spectrum spontaneous SCRS. Key components include a Nd:YAG 532 nm laser emitter for excitation, a 60 × water objective for laser focusing, and an electron‐multiplying charge‐coupled device for collecting SCRS [36]. The FlowRACS instrument is designed with a focus on enhancing the speed and throughput of SCRS and offers two operational modes: profiling and sorting. In this study, we employed the profiling capability of FlowRACS, which, beyond profiling, also enables the deposition of cells as a pool. This pooled sample can subsequently be directly coupled to meta‐sequencing or pool culture, or to single‐cell sequencing or culture after single‐cell encapsulation.

### Analysis of probiotic products by RFC

The quality assessment process using RFC involved the following steps: (i) *Sample Preparation:* A precisely weighed 0.1 g of probiotic powder was dissolved in 900 µL of saline solution. This solution was mixed with 9 mL of saline for a 100‐fold dilution. An equal volume of the probiotic cell dilution was mixed with the Flow‐mode Raman‐activated Cell Sorting Kit (SCC007101), which contains the surfactant PF127 to ensure the uniform dispersion of cells in the liquid phase of microfluidic chips. The mixture was then further diluted (10–100 times) to achieve a suitable concentration of approximately 10^5^ CFU/mL. The resulting mixture was then introduced into a microfluidic chip for RFC detection using an injection pump. (ii) *Detection Setup:* FlowRACS serves as a high‐throughput flow sorter capable of detecting single‐cell metabolic functions in a microfluidic chip system [36]. Calibration was performed before analysis using a silicon wafer within a wavelength range of 320–4256 cm^−1^. For baseline correction, asymmetric reweighted penalized least squares were adopted. Its core lies in iteratively adjusting the weight matrix to suppress baseline drift in spectra signals while preserving characteristic peak information. The smooth parameter was set as 10^6^, and the sensitivity parameter as 0.05 to achieve the deblending aim for each Raman spectrum. Then, the max‐min normalization was utilized to normalize spectra to the [0,1] range without altering peak shapes, thus eliminating intensity fluctuations (like minor laser power changes) and ensuring cross‐batch data comparability and consistent instrument responses. The instrument used is FlowRACS, which is equipped with a 532 nm laser of 300 mW laser power, while a detection time of 1 s per cell was used, with single‐cell profiling or sorting operating at a throughput of 60 events/min.

### Cell count for probiotic products by conventional methods

Cell count was executed using several traditional techniques:

(i) *Hemocytometer Count*: Probiotic products underwent a 1:1000 dilution. A drop of the prepared sample was placed in a hemocytometer chamber, and cells in the corners and middle squares were counted. The results were multiplied by the total number of squares in the chamber and the dilution factor to obtain the total cell count, including both viable and nonviable cells.
(ii) *Plate colony count method:* The probiotic product was serially diluted 10‐fold and cultured on MRS solid agar plates. Each sample was conducted in triplicate to ensure biological duplication, then cultured anaerobically at 37°C for 72 h. The average number of colonies on the plates was recorded and multiplied by the dilution factor to ascertain the total number of live cells, as described previously [31].

### Constructing mock samples, standard samples and probiotic products

A mock multi‐species system was created by mixing *L. plantarum* RH‐LP08 and *B. lactis* BB‐12 in ratios of 1:1, 1:9, 1:99, and 1:999. For the standard sample, which was incorporated as an internal reference during RFC profiling of probiotic samples, *E. coli* DH5α was cultured to the stationary phase, adjusted to the desired concentration, mixed, washed, aliquoted, and stored at −20°C. The concentration was determined microscopically (9.47 ± 1.08 × 10^8^ CFU/mL). Pure deuterium oxide (D_2_O; Sigma‐Aldrich) was used for deuterium labeling. For probiotic products, each sample was incubated with 0.1 mL of 100% D_2_O MRS medium (which was prepared by dissolving MRS medium with D_2_O and then filtering it through a 0.22 μm PES membrane filter) for 3 h, as previously stated [31]. The 100% D_2_O was chosen because the high concentration of D_2_O can (i) accelerate the SCRS profiling process by elevating the intake rate of deuterium, and (ii) also inhibit bacterial growth during D_2_O incubation which can skew subsequent measurement of the ratios among bacterial components [31].

### Data analysis

All Raman spectra underwent preprocessing using the R software of “RamanEx” package. The steps included standardization and centering of the spectra. Machine learning techniques were employed for classification, constructing a database from the Raman spectra of single‐species bacterial powders. To ensure the model's reliability, we employed a 7:3 split for training and validation, which was repeated for five times across four independent batches (each batch was independently prepared and measured on different days to capture potential variations in experimental conditions such as temperature, humidity, and instrument performance). This approach confirmed the model's stability and general applicability across different experimental conditions. A dedicated software suite was developed for processing SCRS, integrating functions such as data quality control (removal of outlier spectra and low signal‐to‐noise ratio spectra), preprocessing (band selection, noise smoothing, baseline correction, and multiple normalization methods), and model construction using various machine learning algorithms, including Random Forest (RF), Linear Discriminant Analysis (LDA), Support Vector Machine (SVM), Naive Bayes Classifier (NBC), K‐Nearest Neighbors (KNN), and Decision Tree (DT).

### Statistical analysis methods

All results from the repeated validations are reported as “mean ± standard deviation.” In this study, for the comparison of differences between two groups of data, the Shapiro–Wilk test was first used to verify whether the data follow a normal distribution. If the data were normally distributed and homogeneity of variance was confirmed, a Student's t‐test was applied; otherwise, the Mann–Whitney *U* test was used.

### LDA implementation

LDA was used as a primary supervised classification technique, aiming to maximize the separation between classes by finding a linear combination of features. Through projecting data onto a new space, LDA maximizes the ratio of between‐class variance to within‐class variance, effectively reducing data dimensionality while preserving discriminative information. LDA's common covariance matrix assumption simplifies computations and reduces overfitting risks compared to more complex classifiers like SVM.

### Calculation of C‐D ratio and MAL

The Raman spectral data of complex probiotic products were predicted using the established model, with the CDR computed as follows: CDR = CD/(CD + CH). This calculation utilized the intensity of the C‐D peak (2040–2300 cm^−1^) and the C‐H peak (2800–3100 cm^−1^) to characterize the degree of deuterium substitution in the C‐H band [38]. The MAL, which indicates cellular vitality, is computed as: MAL = CDR_sample_ − CDR_0h_. CDR values are the averages for samples incubated with 100% D_2_O for 3 h compared to 0 h. A higher MAL indicates greater vitality, defined as: MAL ≤ 0: dead cells; MAL > 0: living cells. The MAL‐Heterogeneity Index (MAL‐HI) is determined by calculating the variance of individual cell MAL values, representing the level of metabolic activity variation among cells. A higher MAL‐HI indicates greater heterogeneity in metabolic activity within the probiotic products. The CDR, MAL, and MAL‐HI calculations were automated through an R script.

## AUTHOR CONTRIBUTIONS

**Jia Zhang**: Conceptualization; methodology; supervision; funding acquisition; visualization; project administration; resources; writing—original draft; writing—review and editing; investigation. **Jianmei Wang**: Methodology; data curation; formal analysis; writing—review and editing; writing—original draft; investigation. **Pengfei Zhu**: Writing—review and editing; methodology; supervision; investigation. **Zhidian Diao**: Writing—review and editing; methodology; data curation; formal analysis; investigation. **Shuhua Tian**: Software; writing—review and editing. **Ziyuan Ding**: Resources; writing—review and editing. **Yongming Duan**: Supervision. **Teng Xu**: Data curation; formal analysis; methodology; investigation. **Xuan Zhou**: Software; formal analysis. **Xixian Wang**: Methodology; conceptualization. **Xia Ma**: Methodology; resources. **Ting Sun**: Supervision. **Xiaoyan Jing**: Methodology. **Weilian Hung**: Resources; validation. **Bo Ma**: Methodology; validation; Supervision; resources. **Shi Huang**: Resources; supervision; writing—review and editing. **Xiaowei Zheng**: Writing—review and editing; resources; methodology; supervision. **Jian Xu**: Writing—review and editing; supervision; methodology; funding acquisition; project administration; conceptualization.

## CONFLICT OF INTEREST STATEMENT

Jian Xu and Bo Ma are among the founders of Qingdao Single‐Cell Biotech, Co., Ltd. Other authors declare no conflicts of interest.

## ETHICS STATEMENT

No animals or humans were involved in this study.

## Supporting information


**Figure S1.** The prediction results between the true proportion of the 15 strains and the proportion results obtained from our predictive analysis.
**Figure S2.** Identification of strains from the same species in mock probiotic samples.
**Figure S3.** Validation of the error rate of our method under varying energy and time conditions.

**Table S1.** Commercial probiotic products used for method assessment in this study.
**Table S2.** Comparison of viability rates for *Bifidobacterium animalis subsp. lactis* YL15, *Lacticaseibacillus paracasei* YL16, and *Lacticaseibacillus paracasei* YL17 using the RFC method versus the traditional methods.

## Data Availability

The data that support the findings of this study are available on request from the corresponding author. The data are not publicly available due to privacy or ethical restrictions. The data and scripts used are saved in GitHub: https://github.com/zhouxuan-dot/250418RFC.git. Supporting materials (figures, tables, graphical abstract, slides, videos, Chinese translated version, and update materials) may be found in the online DOI or iMetaOmics http://www.imeta.science/imetaomics/.
