# Supplementary material for: Raman flow cytometry based single‐cell species classification, viable‐cell counting and vitality test for probiotic products
Source: IMetaOmics. 2025 May 19;2(3):e70024. doi: 10.1002/imo2.70024 (PMC12805996; doi:10.1002/imo2.70024)
Supplement: Supplementary file 1 — Figure S1. The prediction results between the true proportion of the 15 strains and the proportion results obtained from our predictive analysis. Figure S2. Identification of strains from the same species in mock probiotic samples. Figure S3. Validation of the error rate of our method under varying energy and time conditions. [file IMO2-2-e70024-s002.docx]

**Supporting information to Raman Flow Cytometry based single-cell species classification, viable-cell counting and vitality test for probiotic products**

## Running title: Raman Flow Cytometry for probiotic quality assessment

Jia Zhang^1,2,3#,*^, Jianmei Wang^1,3#^, Pengfei Zhu^1,2,4,5#^, Zhidian Diao^1,3#^, Shuhua Tian^4^, Ziyuan Ding^6^, Yongming Duan^6^, Teng Xu^1^, Xuan Zhou^1^, Xixian Wang^1,2,3^, Xia Ma^7,8^, Ting Sun^7,8^ , Xiaoyan Jing^1,2,3^, Weilian Hung^7,8^, Bo Ma^1,2,3^, Shi Huang^9*^, Xiaowei Zheng^6*^, Jian Xu^1,2,3*^

^1^ Single-Cell Center, Key Laboratory of Photoelectric Conversion and Utilization of Solar Energy, Qingdao New Energy Shandong Laboratory, Qingdao Institute of Bioenergy and Bioprocess Technology, Chinese Academy of Sciences, Qingdao, Shandong 266101, China

^2^ Shandong Energy Institute, Qingdao, Shandong 266101, China

^3^ University of Chinese Academy of Sciences, Beijing 100049, China

^4^ Qingdao Single-Cell Biotech. Co., Ltd, Qingdao, Shandong 266101, China

^5^ School of Medicine and Pharmacy, Ocean University of China, Qingdao, 266003, China

^6^ Nutrition & Health Research Institute, COFCO Corporation, Beijing 102209, China

^7^ Inner Mongolia Dairy Technology Research Institute Co., Ltd., Hohhot 010000, China

^8^ Yili Innovation Center, Inner Mongolia Yili Industrial Group Co., Ltd., Hohhot 010000, China

^9^ Faculty of Dentistry, The University of Hong Kong, Hong Kong SAR 999077, China

^#^These authors contributed equally: Jia Zhang, Jianmei Wang, Pengfei Zhu, and Zhidian Diao.

^*^Correspondence to: [xujian@qibebt.ac.cn](mailto:xujian@qibebt.ac.cn) (Jian Xu), [zhangjia@qibebt.ac.cn (Jia Zhang),](mailto:zhangjia@qibebt.ac.cn%20(Jia%20Zhang),) [zhengxiaowei@cofco.com](mailto:zhengxiaowei@cofco.com) (Xiaowei Zheng), and [shihuang@hku.hk](mailto:shihuang@hku.hk) (Shi Huang)

**Supplemental Figures**

**
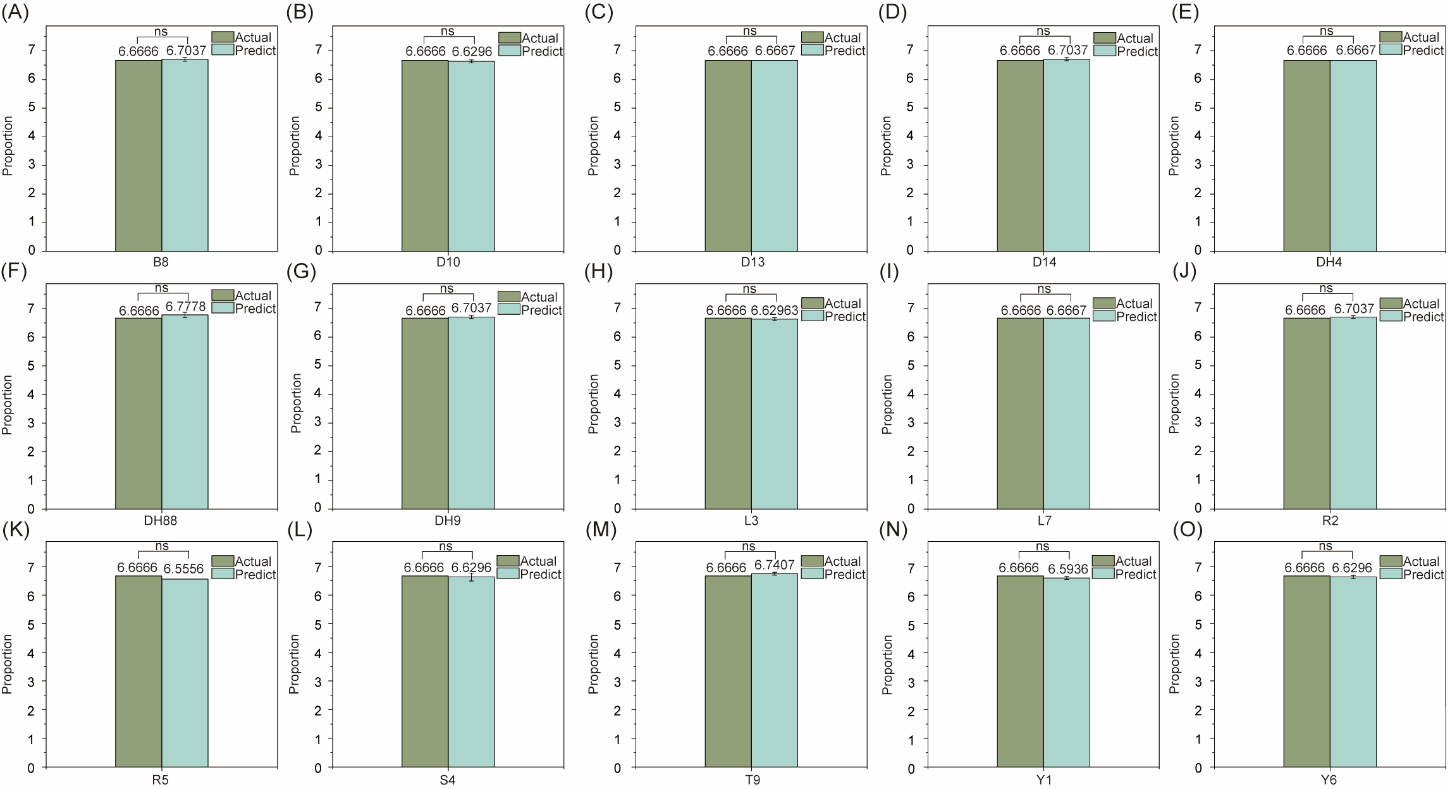
**

**Figure S1.** The prediction results between the true proportion of the 15 strains and the proportion results obtained from our predictive analysis. The 15 strains are categorized as B8 (A), D10 (B), D13 (C), D14 (D), DH4 (E), DH88 (F), DH9 (G), L3 (H), L7 (I), R2 (J), R5 (K), S4 (L), T9 (M), Y1 (N), and Y6 (O), respectively. The proportion is shown as percentage.

**
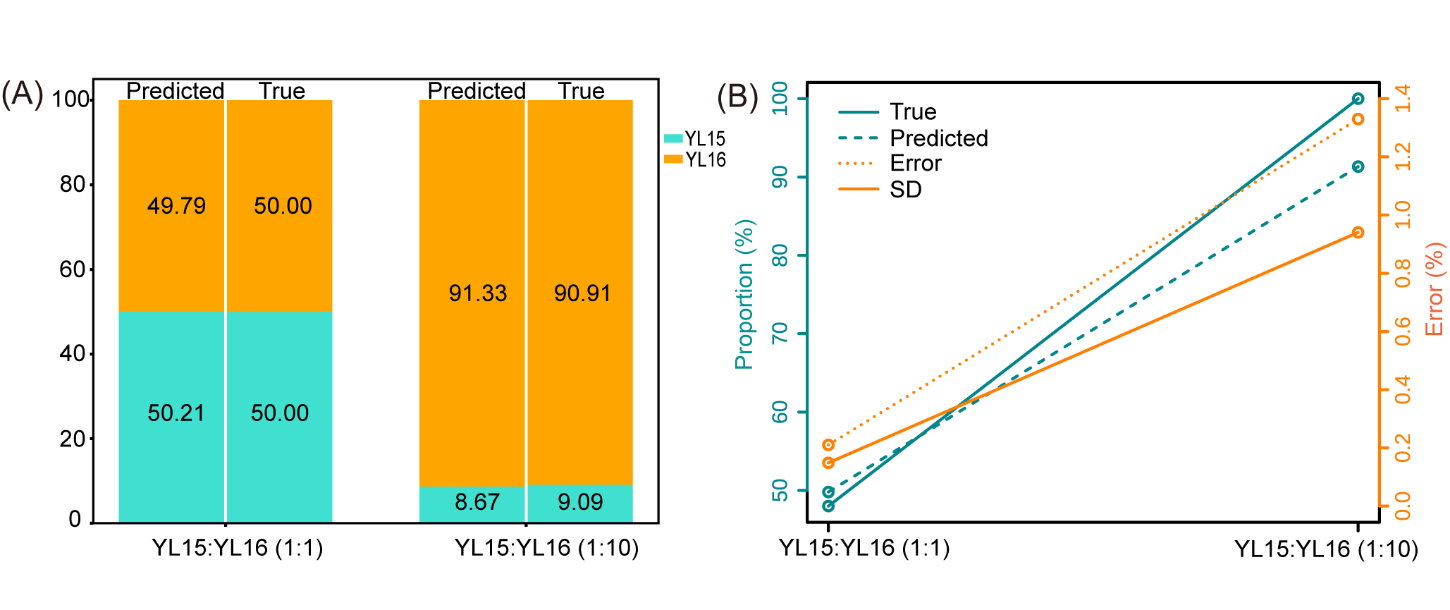
**

**Figure S2.** Identification of strains from the same species in mock probiotic samples. (A) Comparison of performance for predicted versus true relative abundances of mock samples. (B) Proportion and error analysis of predicted versus true relative abundances of mock samples (SD: Standard Deviation).


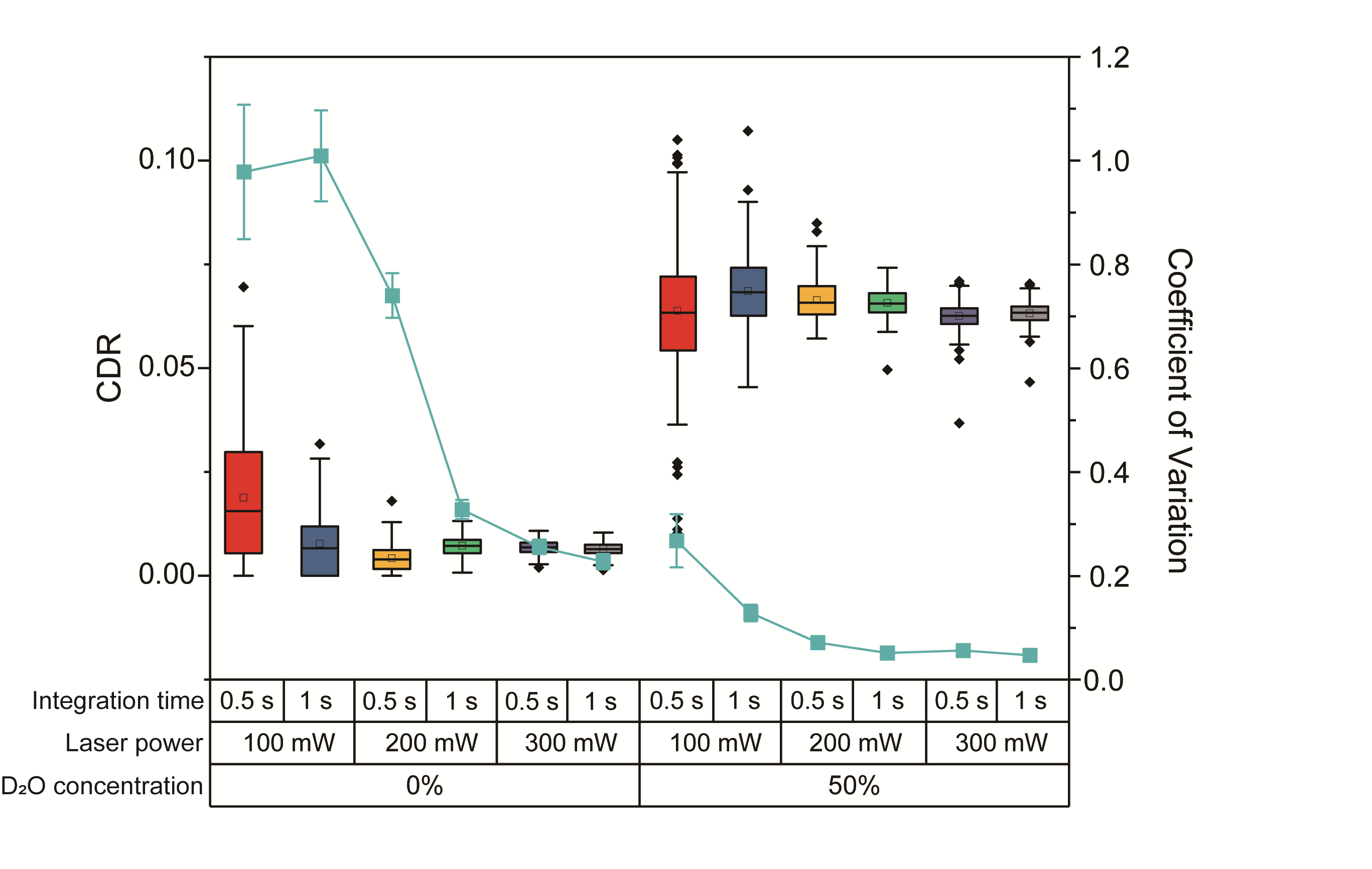


**Figure S3.** Validation of the error rate of our method under varying energy and time conditions. The coefficient of variation (CV) for CDR values (0 and 0.06) of *Escherichia coli* samples was measured for three times under each combination of various energy (100, 200, 300 mW) and acquisition time (0.5 s, 1 s).
